# Child and Adolescent Suicides in Turkey (2004–2023): A Comprehensive Evaluation

**DOI:** 10.1007/s10578-025-01839-x

**Published:** 2025-04-19

**Authors:** Ümit Şimşek, Tuğçen Demircan

**Affiliations:** Department of Forensic Medicine, Faculty of Medicine, Eskişehir Osmangazi University, Eskişehir, Turkey

**Keywords:** Adolescent suicide, Child suicide, Suicide methods, Gender differences, Suicide trends, Forensic medicine

## Abstract

This study analyzes suicide rates, causes, methods, and age- and gender-specific trends among children and adolescents in Turkey (2004–2023) using Turkish Statistical Institute data. Utilizing data from the Turkish Statistical Institute, suicide deaths across two age groups (< 15 years and 15–19 years) were examined. During this period, 8,954 suicides were recorded, with 52.6% involving males. Hanging (41.3%) and firearms (30.2%) were the most frequently used methods, showing significant gender differences (p < 0.01). Family discord emerged as the leading cause of suicide among females, while illness was predominant among males. The findings underscore the urgent need for culturally tailored, evidence-based suicide prevention strategies. Key measures include restricting access to means of suicide, enhancing family-based interventions, and improving mental health services with a focus on early detection. Adoption of the WHO’s “LIVE LIFE Initiative for Suicide Prevention” could provide a robust framework to reduce youth suicide rates in Turkey.

## Introduction

Suicide is a critical public health problem resulting in more than 700,000 deaths worldwide each year [47]. Each suicide has wide-ranging social, emotional, and economic consequences and deeply affects individuals and communities worldwide. Suicide is one of the leading causes of death in children and adolescents in many countries. Therefore, it is important to know the characteristics, causes, risk factors, and methods of child and adolescent suicides.

The World Health Organization (WHO) data indicate that suicide is the fourth leading cause of death globally in the 15–19 year age group [46]. In the United States of America (USA), suicide is the third leading cause of death in the same age group and the second leading cause of death in the 10–14 year age group [8]. In 2019, 51,829 adolescent deaths due to suicides were reported worldwide [25]. The overall suicide-related mortality rate among young people decreased from 4.74 per 100,000 in 1990 to 2.70 in 2019. Notwithstanding the downward trend, the global suicide rate is still high, accounting for 4.2% of total deaths among individuals aged 5–19 years [25, 31]. It has been observed that the suicide rates among children and adolescents have shown different trends across countries over the years. A study examining the global trend of suicide-related deaths in young people between 1990 and 2020 emphasized that suicide rates in many European countries, including Germany, France, Italy, and most of the Scandinavian countries, were on a downward trend. However, suicide rates increased in several central and eastern European countries, including the United Kingdom (UK), Ireland, and Portugal, as well as Poland, Romania, and Russia, the USA, and Australia [4]. In the USA, suicide mortality rates among individuals aged 10–19 years increased by 56% between 2007 and 2016, becoming the second leading cause of injury-related deaths in this age group [11].

Suicide-related deaths in adolescents are directly correlated with biological and psychological developmental processes and socio-cultural changes accompanying the transition from childhood to adulthood [25, 29, 30]. Literature findings indicate that the frequency and characteristics of suicides vary significantly across different stages of adolescence [9, 29, 30]. Moreover, studies have reported that suicide rates among both males and females increase with age. It has also been reported that suicides among older adolescents are often associated with mental disorders. Furthermore, significant variations in suicide methods are observed across different adolescent age groups. While school-related problems and parental conflicts are more prevalent among younger adolescents, relationship problems have been identified as more prominent in older adolescents [9, 27, 29, 30]. These findings highlight the importance of tailoring suicide prevention strategies to address the social and developmental changes that occur during adolescence, rather than treating adolescents as a homogenous group.

Socially, economically, psychologically, and culturally, Turkey is influenced by both Eastern and Western traditions, embodying characteristics of both developed and developing countries. Studies have suggested that suicide rates in Turkey vary significantly over time with respect to age, gender, and methods of suicide [35]. However, despite the seriousness and scope of the issue, national studies on suicides among children and adolescents in Turkey are limited. This has led to a lack of comprehensive understanding regarding the causes, methods, gender and age differences, and temporal trends. Therefore, a detailed examination of the characteristics of suicides in Turkey can provide valuable insights into both national and international epidemiological patterns and contribute to the development of effective suicide prevention strategies.

In this context, this study aims to provide a comprehensive evaluation of the trends in child and adolescent suicide-related mortality rates in Turkey over the past two decades, focusing on gender, age, causes, and methods of suicide.

## Methods

This study was approved by the local ethics committee. Detailed data on suicide deaths in children and adolescents aged < 15 years and 15–19 years between 2004 and 2023, including gender, causes, and methods, were obtained from the Turkish Statistical Institute (TurkStat).

The crude suicide rate was calculated using the formula: (Number of suicides/Total population) × 100,000. Population data were obtained from the Turkish Statistical Institute (TÜİK). This standardized approach allows for temporal comparisons, providing a clearer understanding of suicide trends over time in Turkey.

Reasons for the suicides were classified under the categories of illness, family discord, financial difficulties, romantic relationships, educational failure, other, and unknown. The suicide methods in the TurkStat data were categorized as hanging, chemical poisoning, jumping from a height, drowning, using cutting-piercing instruments, using firearms, self-immolation, using natural gas, jumping under a motor vehicle, and other methods.

The “Suicide Statistics Form” provided by TurkStat is distributed to the police and gendarmerie organizations in provinces and districts. These organizations are responsible for completing the form for each suicide case occurring within their jurisdictions and submitting it to the General Directorate of TurkStat on a monthly basis. TurkStat systematically evaluates and reports these data, ensuring completeness, as every suicide case is mandatorily reported to official institutions.

In Turkey, prosecutors determine whether a suicide has taken place and the nature of the suicide based on the methodology, crime scene investigation, and autopsy report. Reasons for the suicide are reported as the most probable reason in line with the information obtained from family members and close relatives.

Statistical analyses were performed using the SPSS 29 (Statistical Package for the Social Sciences) program. Descriptive statistical methods (mean, standard deviation, frequency, percentage, minimum, and maximum) were used to evaluate the data. The conformity of the quantitative data to a normal distribution was evaluated using the Shapiro–Wilk test and graphical examinations and Student’s t-test was used for comparisons of normally distributed variables between two groups. Pearson’s Chi-square test was used to compare the qualitative data. The statistical significance level was accepted as p < 0.05.

## Results

Over the 20-year period covered by this study, a total of 64,672 suicide cases were recorded in Turkey. Among these, 1,676 cases (2.59%) occurred in individuals under 15 years of age, and 7,278 cases (11.25%) were in the 15–19 year age group. Of the suicide deaths among children and adolescents, 4,717 (52.6%) were male and 4,237 (47.4%) were female.

**Fig. 1:**
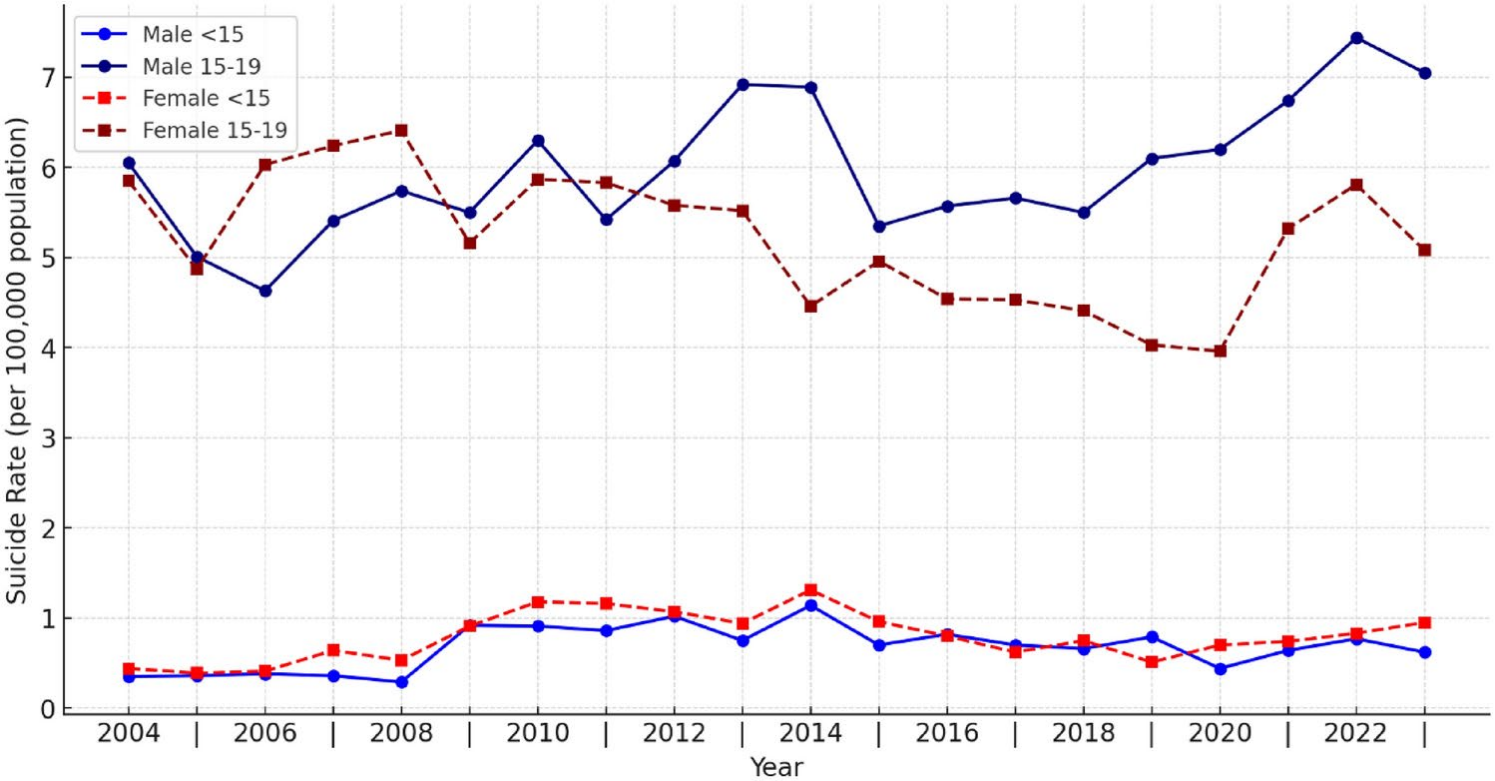
Distribution of suicide rates by age and gender

Crude suicide rates for children and adolescents between 2004 and 2023 are presented in Fig. 1. In the < 15 year age group, the highest suicide rate for males was 1.14 in 2014, while the lowest rate was 0.29 in 2008, with an average rate of 0.67 ± 0.24. For females in this age group, the highest suicide rate was 1.31 in 2014, and the lowest was 0.39 in 2005, with an average rate of 0.79 ± 0.26. The female-to-male suicide rate ratio in the < 15 year age group during the study period was calculated as 1.22. In this age group, crude suicide rates were consistently lower than those observed in the general population.

In the 15–19 year age group, the highest suicide rate for males was 7.44 in 2022, while the lowest was 4.63 in 2006, with an average rate of 5.97 ± 0.72. For females in the same age group, the highest suicide rate was 6.41 in 2008, and the lowest was 3.96 in 2020, with an average rate of 5.16 ± 0.68. The female-to-male suicide rate ratio over the 20-year period in this age group was 0.87. Comparing the two age groups, a statistically significant difference was identified in the crude suicide rates (p = 0.001; p < 0.01). Additionally, crude suicide rates among females in the 15–19 year age group were higher compared to those observed in the general female population.

Table 1 presents the distribution of suicide causes among children and adolescents in Turkey between 2004 and 2023, categorized by age group and gender. The most common cause of suicide among children and adolescents was family discord. During the study period, significant differences were observed between genders and causes of suicide across all age groups (p < 0.01). Family discord was the most frequent cause of suicide among females (13.2%), whereas illness was the leading cause among males (10.6%).

**Table 1:** Causes of adolescent suicide in Turkey: 2004–2023

Cause of suicide *n (%)*	15 Years old			15–19 years old		
	M n = 785	F n = 891	Sig.	M n = 3932	F n = 3346	Sig.
Disease	47 (6)	82 (9,2)	χ^2^:17,297	453 (11,5)	339 (10,1)	χ^2^:107,088
Family discord	70 (8,9)	105 (11,8)	0.008**	342 (8,7)	455 (13,6)	0.001**
Financial difficulties	2 (0,3)	7 (0,8)		104 (2,6)	22 (0,7)	
Romantic relationships	20 (2,5)	29 (3,3)		339 (8,6)	215 (6,4)	
Educational failure	29 (3,7)	23 (2,6)		96 (2,4)	75 (2,2)	
Other	150 (19,1)	137 (15,4)		661 (16,8)	481 (14,4)	
Unknown	467 (59,5)	508 (57,0)		1937 (49,3)	1759 (52,3)	

Bold values indicate statistical significance (*p* < 0.05); italics are used for emphasis where relevant

M: Male; F: Female; Sig.: Significant

Pearson’s Chi-Square Test **p < 0.01

Among girls under 15 years of age, the rate of suicide due to illness was significantly higher than that of males in the same age group (p < 0.05). While the rate of suicide due to romantic relationships was higher among females under 15 years compared to males, this rate increased significantly among males in the 15–19 year age group (p < 0.01). Furthermore, the rate of suicide due to financial difficulties was significantly higher in males than females in the 15–19 year age group (p < 0.01).

In the 15–19 year age group, suicide rates due to illness, financial difficulties, and romantic relationships were significantly higher in males than in females (p < 0.01). On the other hand, suicides due to educational failure were found to be more prevalent among males under 15 years of age (p < 0.05). Among females, suicides related to romantic relationships were significantly more frequent in the 15–19 year age group compared to those under 15 years of age (p < 0.01).

Changes in the preferred suicide methods were observed with increasing age. In the 15–19 year age group, methods such as chemical poisoning, jumping from a height, using firearms, and using a cutting tool were preferred at higher rates compared to the < 15 year age group (p < 0.05). Conversely, the rate of suicide by hanging was significantly higher in the < 15 year age group compared to the 15–19 year age group (p < 0.01) (Table 2).

**Table 2:** Most common methods of suicide in Turkey: 2004 to 2023

The suicide method *n(%)*	< 15 years old			15–19 years old		
	M	F	Sig.	M	F	Sig.
Hanging	472 (60,1)	408 (44,3)	χ^2^:91,761 0.001**	1753 (44,6)	1065 (31,8)	χ^2^:457,596 0.001**
Chemical poisoning	22 (2,8)	100 (10,9)		222**(**5,6)	618 (18,5)	
Jumping from a height	53 (6,8)	121 (13,1)		350 (8,9)	551 (16,5)	
Drowning	6 (0,8)	8 (0,9)		58 (1,5)	37 (1,1)	
Using a firearm	208 (26,5)	247 (26,8)		1347 (34,3)	904 (27)	
Self-immolation	2 (0,3)	0 (0,0)		9 (0,2)	4 (0,1)	
Using a cutting tool	3 (0,4)	2 (0,2)		33 (0,8)	31 (0,9)	
Using natural gas, etc	6 (0,8)	1 (0,1)		20 (0,5)	6 (0,2)	
Jumping under a motor vehicle	1 (0,1)	0 (0,0)		17 (0,4)	12 (0,4)	
Other	12 (1,5)	34 (3,7)		123 (3,1)	118 (3,5)	

Bold values indicate statistical significance (*p* < 0.05); italics are used for emphasis where relevant

M: Male; F: Female; Sig.: Significant

Pearson’s Chi-Square Test **p < 0.01

**Fig. 2:**
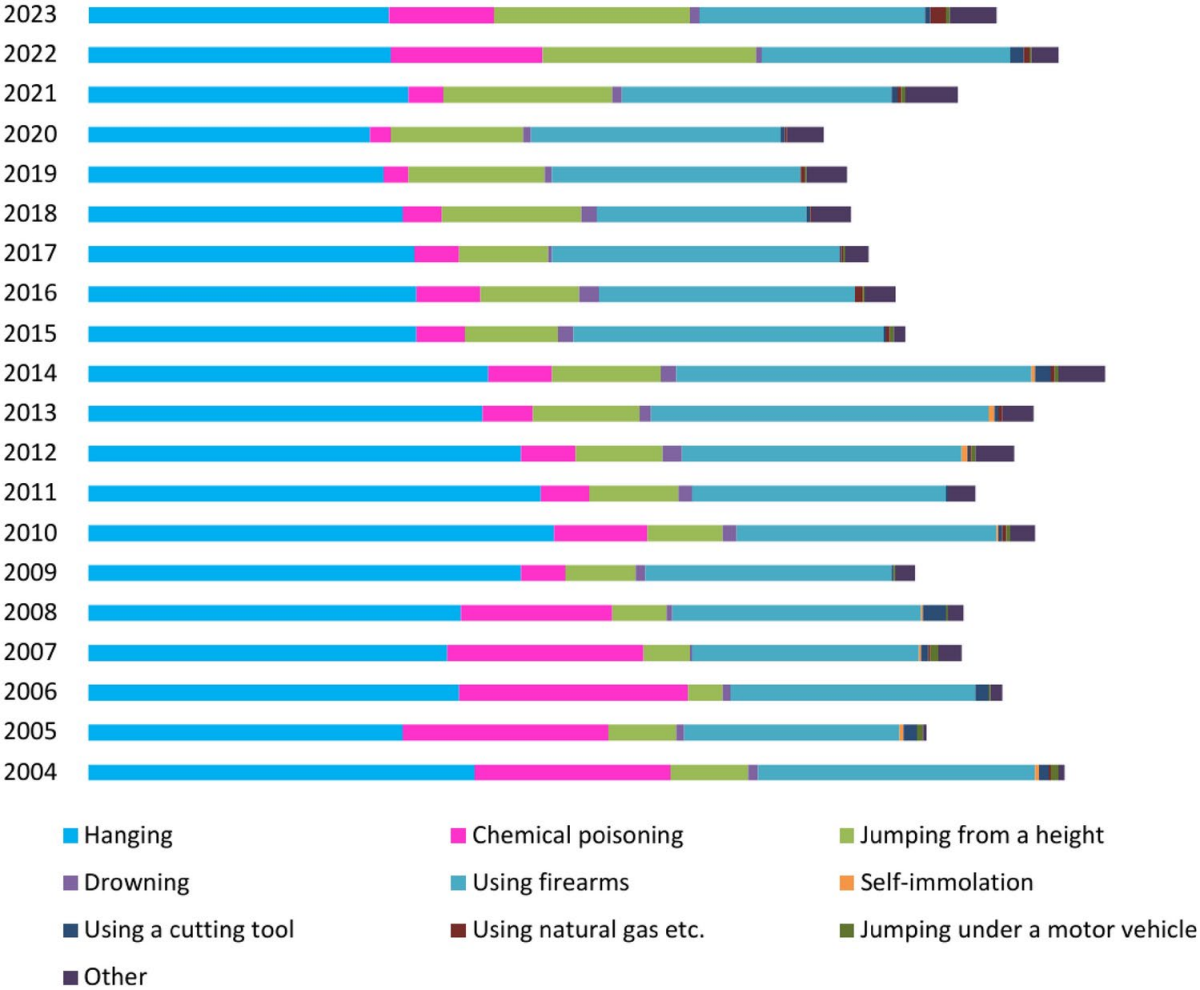
Distribution of suicide methods by years

The data also revealed significant changes in suicide methods over the years. While hanging and firearms remained the most common suicide methods, an increase was observed in other methods such as jumping from a height and chemical poisoning (Fig. 2).

## Discussion

This study is one of the most up-to-date descriptive studies examining child and adolescent suicide rates and trends in Turkey based on the official mortality database of TurkStat. Our study comprehensively analyzed child and adolescent suicides in Turkey between 2004 and 2023 in terms of gender, age groups, methods, and causes. Suicides were predominantly concentrated in the 15–19 year age group, with hanging and firearms identified as the most frequently used methods. Significant gender differences were observed, with males showing a higher overall suicide rate, while family discord and illness were prominent causes among females and males, respectively. These findings underline the importance of understanding age- and gender-specific trends to develop culturally tailored and effective prevention strategies.

Our results present significant gender and age-related differences in line with global trends but also reveal cultural characteristics specific to Turkey. International differences in child and adolescent suicide rates and methods [9, 26, 27, 29] may be explained by variations across countries in sociodemographic, health, and cultural factors, as well as differences in prevention efforts and accessibility of suicide tools. In a meta-analysis using the WHO's mortality database from 45 mostly high-income and middle-income countries, covering the period from 2010 to 2018, it was reported that the lowest suicide rate among 10–19-year-olds was in Israel (1.31 per 100,000), while the highest suicide rate was in Estonia (9.72 per 100,000) [17]. While the USA has the seventh highest suicide rate among young people globally (5.91 per 100,000), the UK has the seventh lowest suicide rate (2.35 per 100,000) [17]. Our data indicated that the suicide rate in Turkey (3.17/100 000) at the age of 19 years and younger is higher than the suicide rate in many other countries. Most European countries (except the UK) have reported decreasing rates of suicide in children and adolescents, whereas increasing rates have been observed in the USA, as well as in most of Central Latin America and Australia [4, 28] Based on our analysis, there was no significant decrease in child and adolescent suicide rates in Turkey. On the contrary, an increase was recorded after 2020, especially in the 15–19 year age group. It is noteworthy that this period coincides with the COVID-19 pandemic and its aftermath. Recent studies have suggested a significant increase in suicide risk among adolescents during the pandemic. One meta-analysis study reported that suicidal thoughts (10.8%) and suicide attempts (4.68%) became more common during the pandemic compared to the period before it [14]. Another meta-analysis study suggested that problems on the suicide spectrum increased among young individuals during the pandemic and that there was a 10% increase in suicide deaths [3]. These results suggest that factors such as social isolation, academic pressure, economic uncertainties, and limited access to mental health services may have increased the risk of suicide among children and adolescents during the pandemic. The increasing trend in Turkey is also consistent with global data and reveals that the pandemic represented a critical period that negatively affected mental health in this age group.

Adolescent suicide deaths are much more common in males than in females (2–5 times more common) [9, 26, 28]. The major exceptions to this gender difference have been observed in China, India [33], and Uzbekistan [17]. In our study, we found that 52.6% of suicide deaths in children and adolescents were male and 47.4% were female. However, our findings revealed that the male-to-female suicide rate ratio was 0.82 among adolescents younger than 15 years but increased to 1.15 in the 15–19 year age group. The higher suicide rates in females < 15 years of age compared to males may challenge the current paradox of suicidal behavior in which death rates from suicide are lower in females compared to males, although females have higher rates of suicidal ideation and attempts compared to males [39]. One of the reasons for the higher suicide mortality rates in females aged < 15 years and the lower male/female ratio in the 15–19 year age group compared to the rates reported in the literature may be explained by the fact that the patriarchal structure and intergenerational and gender role conflicts that cause cultural value tension for women are more prominent in our country compared to Western countries.

Gender-based social norms and patriarchal structures in Turkey significantly impact the mental health of adolescent girls. Prior studies have demonstrated that early marriage, rigid gender expectations, and societal pressures contribute to heightened psychological distress, depression, and suicidal ideation among young females [7]. Adolescent girls frequently experience family-imposed restrictions, educational limitations, and economic dependence, which exacerbate emotional and psychological challenges [7]. These structural inequalities further restrict access to education, financial independence, and mental health services, increasing vulnerability to distress and hopelessness.

The result that suicide rates were significantly higher in the 15–19 year age group compared to the group under the age of 15 years is consistent with studies examining the course of suicidal thoughts and behaviors during adolescence. While suicidal ideation is rare in childhood, it displays a significant increase in the transition to adolescence [18, 34]. In the transition from early adolescence to late adolescence, susceptibility to suicidal behavior increases. The increase in suicidal thoughts and behaviors during adolescence overlaps with increases in the rates of psychopathology types such as depression, anxiety disorder, self-harm, and substance abuse that carry a suicide risk [18, 34]. Moreover, the low suicide rate observed during preadolescence may be attributed to children's cognitive limitations in understanding the concept of death and their underdeveloped ability to plan suicidal acts [32].

### Causes of Suicide

Based on our analysis, the most common cause of suicide among children and adolescents in Turkey was family discord. Family structure plays a critical role in the ability of adolescents to cope with difficulties. Many studies have reported that various risk factors related to family structure and processes are associated with suicidal behavior [43, 48]. Factors such as the death of a family member, divorce, lack of communication, or domestic abuse are the main factors that may increase the risk of suicide in adolescents. A systematic review revealed that parent-adolescent conflict is the most common triggering factor for suicidal behaviors in adolescents [43].

One of the major challenges in analyzing suicide causes among children and adolescents is the high proportion of cases with unknown causes. In our dataset, more than half of the suicide cases lacked a specified cause, restricting the ability to establish definitive risk factor correlations and limiting the potential for targeted preventive interventions. The high percentage of missing data may stem from multiple factors, including inconsistencies in data reporting, sociocultural stigma surrounding suicide, family reluctance to disclose sensitive information, and inherent limitations in forensic classification processes [15, 44].

Several epidemiological studies have indicated that the majority of individuals who attempt suicide have a diagnosable mental disorder and have highlighted that mental disorders are among the strongest predictors of suicide attempts [18, 34]. Psychological autopsy studies have revealed that more than 90% of suicide cases in adolescents are associated with comorbid mental disorders [37, 42]. The increasing prevalence of mental health problems during adolescence, combined with psychosocial risk factors, heightens adolescents' vulnerability to suicidal behaviors. These psychosocial risk factors include depression, substance abuse, anxiety, aggression or impulsivity, family and school problems, and exposure to traumatic life events [37, 42, 48]. In our study, illness was found to be the second most common cause of suicide. However, the extent of the mental illnesses mentioned among the reasons for suicide in Turkey could not be clearly evaluated since the disease category was not subdivided into subgroups in the TurkStat data.

We found that the rates of suicide due to financial difficulties were higher among males aged 15 to 19 years compared to females. This result is consistent with some studies suggesting that economic difficulties may lead to more negative mental health outcomes in males. For example, a large international study reported that economic inequality was significantly associated with depressive symptoms in males, but not in females [48]. Similarly, studies conducted in the USA have indicated that males attending low-income schools have a higher risk of suicidal ideation and attempts, but the same effect is not observed in females [16].

Previous studies have revealed that suicide triggers vary with age. Suicide under 15 years is often linked to family conflicts, maltreatment, or educational failure, while romantic relationships and psychiatric disorders are more common risk factors in those aged 15–19 years [9, 20, 27, 30]. Our study identified educational failure as the third leading cause of suicide among children under 15 years. Seasonal trends were also noted in other studies, such as lower suicide rates during summer vacations in Sweden and Austria, attributed to reduced school stress [13, 24]. In the 15–19 year age group, suicides related to romantic relationships increased, reflecting cultural and socioeconomic differences in risk factors. The reluctance of males to express emotional problems due to fear of being perceived as weak, combined with instances where suicide has been used as a means to punish their partners, may lead to the adoption of more lethal methods [41].

### Methods of Suicide

Similarities in the accessibility and lethality of suicide means have led to the clustering of specific suicide method patterns in some countries. In a suicide method cluster analysis covering 101 countries (2000–2009) and focusing on the 10–19 year age group, it was shown that many countries were in a cluster dominated by hanging [29]. Hanging is the most common method of suicide in countries such as the UK [49], Ireland [2], Canada [40], and Mexico [1]. Moreover, hanging is the most commonly used method among children under 12 years of age in the USA [6]. A recent comprehensive international review confirmed the high prevalence of hanging in 48%-90% of child suicides [12]. These results are compatible with our study. In our study, the most common method of suicide was hanging (41.3%), followed by using firearms (30.2%), jumping from a height (12%), and chemical poisoning (10.7%).

In Turkey, 27.1% of female children and adolescents and 32.9% of male children and adolescents used firearms as a method of suicide. The rate of suicide by firearm among those aged 15–19 years was higher than among those under 15 years of age. At the international level, the use of firearms has been the second most common method of suicide among young people [12]. In contrast to many other countries, firearms are the most common method of suicide among young people in the USA, accounting for 43.6% of all suicide deaths among 10–19-year-olds between 2016 and 2020, followed by hanging/drowning (42.7%) and self-poisoning (6.8%) [23]. In Turkey, the minimum age for obtaining a firearm license is 18 years [45]. Considering that those under this age cannot obtain a license, the high rate of suicide by firearms may be related to inadequate parental supervision and control measures regarding access to firearms. Storing firearms unloaded, keeping them in locked locations, and securing ammunition in a separate locked location from the firearm are measures associated with a reduction in firearm-related suicides [21].

Suicide by jumping from a height has been observed to gradually increase among females over the years. A study conducted in Turkey identified an increasing trend in this method among females aged 15 to 44 years [19]. This rise may be attributed to the widespread use of popular locations for suicide, the increased tendency of individuals with psychiatric disorders to resort to this method in hospital or home settings, and restricted access to other means [22]. Similarly, studies from Singapore and Hong Kong reported that jumping from a height was the most common method of suicide, likely due to the prevalence of high-rise buildings in these regions [5, 36]. As one of the most frequently used methods of suicide, jumping from a height requires targeted attention in suicide prevention strategies. Evidence suggests that restricting access to suicide hotspots and implementing prevention measures are effective in reducing suicide by jumping [5, 10, 36]. Media reports on suicide by jumping have been reported to be an important risk factor for the increase in subsequent suicide cases, and it has been emphasized that guidance on responsible media reporting is necessary [32].

A significant difference was found in suicide by poisoning according to gender differences in a study examining international suicide-related mortality data in young people aged 10–19 years. Males were found to commit suicide by poisoning more than females [17]. Our study revealed that females more frequently chose poisoning by chemical substances as a suicide method compared to males. However, comparative studies have indicated that the rate of drug poisoning is higher among females, whereas the rate of poisoning by other substances is more prevalent among males [17, 38]. While the present study was not able to examine the differences according to the source of poisoning, this issue represents an important direction for future research.

### Limitations

The results of this study have a number of limitations. The TurkStat data includes only officially recorded suicide cases, and the possibility of unreported or misclassified suicide cases cannot be ruled out. The causes and methods of suicide were categorized under general headings, but detailed subcategories within these classifications are missing. For instance, the category “illness” does not distinguish between mental and physical illnesses.

Another limitation of this study is that the reasons for suicide could not be determined in 4,671 cases (52.1%). This significant proportion of unknown causes limits the ability to comprehensively analyze and interpret the underlying risk factors associated with suicides.

Additionally, the study’s cross-sectional design limits the ability to analyze causal relationships or provide an in-depth examination of the reasons behind changes over time. The cultural, religious, and social contexts that influence suicidal behaviors were not sufficiently explored, which may restrict the generalizability of the findings to other contexts. Furthermore, the absence of psychological autopsy methods prevented a more detailed analysis of individual-level risk factors, such as personal history, mental health conditions, or situational triggers, which could have provided deeper insights into the complexities of suicidal behaviors.

## Conclusion

This study provides significant insights through a comprehensive analysis of suicide rates, causes, methods, and demographic characteristics among children and adolescents in Turkey. The findings reveal that suicides were predominantly concentrated in the 15–19 year age group, highlighting this period as a critical risk phase for young individuals. Factors such as illness and family discord were identified as key contributors to suicides in this population.

In an international context, suicide rates in Turkey are higher than in many other countries. Hanging and firearms emerged as the most frequently used methods, emphasizing the importance of limiting access to means of suicide. Policies to restrict access to firearms and ensure safe storage should be implemented, alongside protective measures for high-risk locations such as high-rise buildings. Family-focused counseling programs and school-based psychological support services that promote emotional resilience in young people are essential.

The WHO’s “LIVE LIFE Initiative for Suicide Prevention” outlines four key evidence-based strategies: limiting access to means of suicide, raising public awareness, promoting responsible media reporting, and strengthening mental health services. Adopting these strategies in Turkey could create a sustainable framework for reducing child and adolescent suicide rates.

Responsible media reporting and stronger controls on the dissemination of suicide-promoting content on social media are vital to prevent suicide contagion. Further research is needed to explore the underlying causes and methods of suicide, including psychological autopsy studies, to better understand the complexities of suicidal behavior.

Tracking trends in youth suicide over time will guide health policy and prevention efforts. The comprehensive adoption of WHO’s recommended strategies can enhance the effectiveness of national and local initiatives aimed at reducing suicide rates among children and adolescents.

## Data Availability

The data that support the findings of this study were obtained from the Turkish Statistical Institute (TÜİK) official website (https://www.tuik.gov.tr/). The data are publicly available and can be accessed through the relevant sections of the website.
